# Does Source of Admission Matter? Hospital Outcomes of Older Adults Requiring Nursing Care Hospitalized in Internal Medicine Departments

**DOI:** 10.3390/medicina62101880

**Published:** 2026-09-28

**Authors:** Galina Plotnikov, Natali Babaganov, Nira Koren-Morag, Ahmad Afana, Dana Kagansky, Miya Sharfman, Yochai Levy, Nadya Kagansky

**Affiliations:** 1 Shamir (Assaf Harofeh) Medical Center, Beer Yaakov 7033001, Israel; 2 Gray Faculty of Medical and Health Sciences, Tel Aviv University, Tel Aviv 6997801, Israel; nira.morag@gmail.com (N.K.-M.);; 3 Shmuel Harofeh Geriatric Medical Center, Beer Yaakov 7030803, Israelmiya.sharfman@gmail.com (M.S.)

**Keywords:** older adults, nursing homes, home care, internal medicine, hospitalization, mortality, long-term care

## Abstract

*Background and Objectives*: Older nursing-care patients are a clinically complex population with marked functional dependence, cognitive impairment, nutritional vulnerability, and multimorbidity. Although nursing-home residents are often considered at higher risk for adverse outcomes, many highly dependent older adults receive nursing care at home. *Materials and Methods*: This single-center retrospective cohort study included 501 nursing-care patients hospitalized between December 2022 and December 2023, of whom 334 were admitted from home and 167 from nursing homes. The primary outcome was in-hospital mortality; secondary outcomes included length of hospital stay, discharge destination, and 90-day post-discharge mortality among patients discharged alive. Longer-term survival analyses were exploratory. Demographic, clinical, functional, nutritional, laboratory, hospitalization, and mortality data were evaluated. *Results*: The median age was 85 years (IQR, 11), and 63.1% of patients were women. Patients admitted from home were older than those admitted from nursing homes (median 86 vs. 84 years; *p* = 0.036). Nursing-home patients more frequently presented with fever and infection-related diagnoses and had a higher prevalence of dementia (85.0% vs. 58.1%; *p* < 0.001), greater pressure-ulcer risk according to the Norton scale (*p* < 0.001), and greater nutritional vulnerability according to the MNA-mini (*p* = 0.007). Median length of stay did not differ significantly between groups (*p* = 0.755). In-hospital mortality was 23.7% in patients admitted from home and 18.6% in those admitted from nursing homes (*p* = 0.194). Among patients discharged alive, 90-day post-discharge mortality was 24.3% and 28.7%, respectively (*p* = 0.348). In the extended Cox model for 90-day post-discharge mortality, neither source of admission nor its time-dependent interaction was significantly associated with mortality (source of admission: HR, 0.831; 95% CI, 0.097–7.096; *p* = 0.865; time-dependent interaction: HR, 1.036; 95% CI, 0.573–1.873; *p* = 0.907). *Conclusions*: No statistically significant difference in in-hospital mortality was detected according to source of admission. Nursing-home patients had greater cognitive, nutritional, pressure-ulcer-risk, and infection-related vulnerability. These findings do not establish equivalence between groups and should be interpreted together with individual clinical characteristics.

## 1. Introduction

In the United States, the population aged 65 years and older continues to grow rapidly. According to the U.S. Administration for Community Living, 57.8 million Americans were aged 65 years and older in 2022, representing 17.3% of the total U.S. population, or more than one in every six Americans [1]. Federal aging indicators published in 2024 further confirm the continued growth of the older population and its increasing health-care needs [2]. In 2022, approximately 1.3 million Americans aged 65 years and older lived in nursing homes; the proportion increased substantially with age, from about 1% among those aged 65–74 years to 3% among those aged 75–84 years and 8% among those aged 85 years and older [1]. Older adults also account for a disproportionate share of acute hospital utilization in the United States, with HCUP data showing that adults aged 65 years and older account for more than one-third of U.S. community hospital stays [3,4].

A similar demographic trend is observed in Israel. At the end of 2023, approximately 1.252 million people aged 65 years and older comprised 12.6% of the population. About 4% of this age group, approximately 52,000 people, lived in institutional settings, including nursing homes, assisted-living facilities, hospitalization institutions, and hostels [5]. A substantial proportion of older adults also require community-based support, with a relatively high share of long-term care provided at home compared with other OECD countries [6,7]. Although people aged 65 years and older constitute only about one-eighth of the population, they account for a disproportionately large share of hospital utilization. Previous data show that older adults account for a substantial proportion of patients hospitalized in internal medicine wards and contribute considerably to hospital bed utilization and resource use [8,9].

Acute medical wards frequently care for older patients with severe functional dependence, dementia, multimorbidity, and high clinical vulnerability. These patients, including nursing-home residents and bedridden older adults, are at increased risk for hospital-associated complications, functional decline, prolonged hospitalization, and mortality [9,10,11,12].

A study conducted at a single medical center found that elderly bedridden patients with dementia accounted for a substantial proportion of resources in internal medicine wards. In that study, 24.3% of patients hospitalized in internal medicine wards were elderly bedridden patients with dementia; among patients aged 85 years and older, approximately 46% were bedridden, and 39.3% were carriers of multidrug-resistant organisms [9].

A longitudinal study from Taiwan found that a substantial proportion of older adults experienced functional disability during the last year of life, emphasizing the close association between functional decline, institutional care, and end-of-life trajectories [13].

International studies have reported variable hospitalization outcomes among patients admitted from long-term care facilities compared with community-dwelling older adults [14,15]. A limitation of many such comparisons is that the community group may include relatively independent older adults, whereas nursing-home residents generally have greater baseline dependency and care needs. Less is known about outcomes when the home-based comparison group is itself composed of patients already requiring substantial nursing care. This distinction is clinically important because it reduces, although does not eliminate, differences in baseline dependency between care settings.

In Israel, a substantial proportion of long-term nursing care is provided in the community rather than in institutions [6,7]. Among highly dependent patients, differences in caregiver availability, care-transition pathways, and access to long-term care may plausibly influence hospitalization and discharge processes; however, such mechanisms should be considered hypotheses unless directly measured. Recent evidence from internal medicine wards has linked delayed discharge with advanced age, dependence in activities of daily living, caregiver absence, and the need for long-term care placement [16]. In the present study, therefore, the home group represents nursing-care-dependent patients living in the community rather than independent community-dwelling older adults.

Therefore, this study aimed to examine whether source of admission was associated with mortality among nursing-care-dependent patients aged 65 years and older admitted to internal medicine departments from home or from standard nursing-home facilities, while accounting for individual clinical characteristics.

## 2. Methods

### 2.1. Study Design and Setting

This was a retrospective cohort study conducted at a single medical center. Electronic medical records of nursing-care-dependent patients hospitalized in internal medicine departments between December 2022 and December 2023 were reviewed. Study eligibility was based on the Israeli Ministry of Health (MoH) ‘nursing’ (si’udi) functional category, characterized by severe and persistent limitations in mobility and basic activities of daily living and a need for prolonged nursing care and monitoring [17]. The same eligibility criteria were applied to patients admitted from home and from nursing homes. Nursing-care status was obtained from the electronic medical record, and a geriatric specialist additionally reviewed the records to confirm eligibility. Patients admitted from complex nursing-care facilities or chronic ventilation units were excluded because these settings represent distinct care categories with greater medical complexity. Patients who did not meet nursing-care dependency criteria were also excluded.

Clinical information was obtained from the hospital electronic medical records. Variables included age, sex, residence, chief complaint at admission, main diagnosis, chronic diseases, pressure wounds present on admission, nursing-care dependency documentation, nutritional and pressure-ulcer risk assessments, discharge destination, and mortality outcomes. Laboratory measurements were obtained at admission or during the first two days of hospitalization. If multiple measurements were available during this period, the first recorded value was used for the analysis. Chief complaints were grouped into six clinically meaningful categories because some of the original categories had small sample sizes, which could have led to unstable comparisons. The final groups were: fever, shortness of breath, confusion, laboratory abnormalities, general deterioration, and other. Main diagnoses were also grouped according to clinical relevance: pneumonia, urinary tract infection (UTI), other infection, CVA/TIA, and other diagnoses.

Comorbidity burden was assessed using the Age-adjusted Charlson Comorbidity Index (ACCI) and analyzed both as a continuous score and as severity categories: 2–4 = mild, 5–6 = moderate, and ≥7 = severe [18].

Pressure-ulcer risk was assessed using the Norton scale and analyzed both as a continuous score and as severity categories: ≥19 = mild risk, 15–18 = moderate risk, and ≤14 = severe risk [19].

Nutritional risk was assessed using the Malnutrition Universal Screening Tool (MUST) numeric score. The Mini Nutritional Assessment short form (MNA-mini) was analyzed as a continuous score [20,21,22]. MUST categories were not used in the reported group comparisons and therefore are not defined here.

### 2.2. Outcomes

The primary outcome was in-hospital all-cause mortality. Secondary outcomes were length of hospital stay, discharge destination, and 90-day post-discharge mortality among patients discharged alive. Longer-term time-to-event analyses were considered exploratory and are reported separately from post-discharge 90-day survival to avoid combining outcomes with different at-risk populations and time origins.

Additional descriptive analyses compared demographic, clinical, nutritional, pressure-ulcer-risk, comorbidity, laboratory, admission-presentation, and main-diagnosis characteristics between patients admitted from home and those admitted from nursing homes.

Length of stay was calculated from hospital admission to discharge or in-hospital death. Because in-hospital death precludes live discharge, length of stay should be described using its full distribution and interpreted with death as a competing event when time to live discharge is analyzed. Discharge destination categories were original location, complex nursing care, chronic ventilation, and other destinations.

### 2.3. Statistical Analysis

Continuous variables were tested for normality using the Kolmogorov–Smirnov test. Normally distributed variables were summarized as mean ± standard deviation and compared using independent-samples *t* tests; non-normally distributed variables were summarized as the median (interquartile range [IQR]) and compared using the Mann–Whitney U test. Categorical variables were presented as counts and percentages and compared using Pearson’s chi-square test; Fisher’s exact test was used when expected cell counts were small. Kaplan–Meier curves were used to describe all-cause survival from hospital admission and 90-day post-discharge survival among patients discharged alive, with survival distributions compared using the log-rank test. For the all-cause survival analysis, follow-up was calculated from hospital admission until death or administrative censoring. The administrative censoring date was 13 February 2026. Multivariable Cox regression was used for time to all-cause death and for death within 90 days after discharge. Covariates were selected a priori on the basis of clinical relevance rather than univariable statistical significance. The proportional hazards assumption was initially assessed by visual inspection of partial residual plots and log-minus-log survival curves. Time-dependent covariates, defined as interactions between each covariate and ln(time), were subsequently introduced into the Cox models to formally assess time-varying effects. Significant time-dependent interaction terms (*p* < 0.05) indicated violation of the proportional hazards assumption for several covariates; therefore, extended Cox models incorporating time-dependent covariates were fitted. All tests were two-sided, and *p* < 0.05 was considered statistically significant. All statistical analyses were performed using IBM SPSS Statistics, version 31.0 (IBM Corp., Armonk, NY, USA).

### 2.4. Sample Size Calculation

Assuming a 90-day mortality rate of approximately 20%, group sizes of 334 and 167 patients provide 80% power, at a two-sided significance level of 0.05, to detect an absolute between-group difference of approximately 12 percentage points using a two-sample comparison of independent proportions.

## 3. Results

### 3.1. Study Population and Baseline Characteristics

During the study period, 501 nursing-care patients were admitted to internal medicine wards: 334 from home and 167 admitted from nursing homes. Overall, 316 patients (63.1%) were women. The proportion of women was higher among patients admitted from home than among those admitted from nursing homes, although the difference was not statistically significant. The median age of the cohort was 85 (11) years. Patients admitted from home were older than those admitted from nursing homes. The participant selection process is summarized in Figure 1.

The distribution of main complaints differed significantly between the groups. Fever was more common among patients admitted from nursing homes, whereas general deterioration was more common among patients admitted from home. The distribution of main diagnoses also differed significantly: pneumonia and UTI were more common among nursing-home patients, while other infections, CVA/TIA, and other diagnoses were more frequent in the home group.

ACCI severity was high in both groups with no statistically significant difference between groups. Norton severity differed significantly between groups: severe Norton risk was present in 98.2% of patients admitted from nursing homes compared with 89.5% of those admitted from home (Table 1).

### 3.2. Comorbidities and Risk Factors

Most comorbidities were similarly distributed between the two groups. Diabetes mellitus was more frequent among patients admitted from home than those admitted from nursing homes (43.4% vs. 28.1%, *p* < 0.001). Dementia was significantly more common among patients admitted from nursing homes (85.0% vs. 58.1%, *p* < 0.001). Pressure wounds on admission were numerically more common among patients admitted from nursing homes, but this difference did not reach statistical significance (51.5% vs. 43.1%, *p* = 0.076) (Table 2).

### 3.3. Pressure-Ulcer Risk, Nutritional, Comorbidity, and Laboratory Measures

Patients admitted from nursing homes had lower median (IQR) Norton scores than those admitted from home, indicating greater pressure-ulcer risk and lower median (IQR) MNA-mini scores, indicating greater nutritional vulnerability. The median MUST score and median (IQR) ACCI score did not differ significantly between groups. Among laboratory variables, patients admitted from nursing homes had higher WBC, neutrophil count, and hemoglobin values (Table 3).

### 3.4. Hospitalization Outcomes and Mortality

In-hospital mortality occurred in 110 of 501 patients (22.0%) and did not differ significantly between patients admitted from home and those admitted from nursing homes (23.7% vs. 18.6%; *p* = 0.194). For the long-term all-cause mortality analysis, deaths occurring from hospital admission through the end of the study follow-up period were included. Using this definition, 406 deaths were recorded during follow-up. All-cause mortality during follow-up did not differ significantly between groups (80.5% vs. 82.0%; *p* = 0.687). Among the 391 patients discharged alive, 101 (25.8%) died within 90 days after discharge; 90-day post-discharge mortality was 24.3% in the home group and 28.7% in the nursing-home group (*p* = 0.348).

Median length of hospital stay was 7.0 days (IQR, 9.0) in the overall cohort and did not differ significantly between patients admitted from home and those admitted from nursing homes (*p* = 0.755). Discharge destination among hospital survivors also did not differ significantly between groups (global *p* = 0.104) (Table 4).

Kaplan–Meier analysis of 90-day post-discharge survival included 391 patients discharged alive: 255 admitted from home and 136 admitted from nursing homes. There were 101 deaths within 90 days after discharge (62 in the home group and 39 in the nursing-home group). Mean estimated survival was 77.2 days (95% CI, 74.0–80.4) in the home group and 72.7 days (95% CI, 67.5–77.9) in the nursing-home group. Median survival was not reached in either group. The survival distributions did not differ significantly (log-rank *p* = 0.277) (Table 5 and Figure 2).

### 3.5. Total Survival Analyses

Exploratory time-to-event analyses were performed for all-cause mortality from hospital admission. Kaplan–Meier analysis included all 501 patients, of whom 406 died, and 95 were censored. The median follow-up time was 174 days (95% CI, 125–223). The administrative censoring date was 13 February 2026. Mean estimated survival was 414 days (95% CI, 367–462) in the home group and 350 days (95% CI, 289–412) in the nursing-home group. Median survival was 198 days (95% CI, 133–263) and 150 days (95% CI, 95–205), respectively. The survival distributions did not differ significantly (log-rank *p* = 0.183). (Supplementary Table S1 and Supplementary Figure S1).

### 3.6. Extended Cox Regression Analysis

Extended Cox regression models incorporating time-dependent covariates were fitted for all-cause mortality and 90-day post-discharge mortality. For all-cause mortality from hospital admission (Supplementary Table S2), the model included 493 patients, with 398 deaths and 95 censored observations; eight patients were excluded because of missing covariate data. Significant time-dependent interactions were observed for age, neutrophil count, albumin, dementia, and source of admission, indicating that their associations with all-cause mortality varied over time. For source of admission, both the main term (HR, 4.361; 95% CI, 2.127–8.940; *p* < 0.001) and the interaction with ln(time) (HR, 0.713; 95% CI, 0.616–0.827; *p* < 0.001) were significant, indicating a time-varying association with all-cause mortality.

For 90-day post-discharge mortality among hospital survivors (Supplementary Table S3), the extended Cox model included 388 patients, with 100 deaths and 288 censored observations; three patients were excluded because of missing covariate data. Significant time-dependent interactions were observed for age, neutrophil count, and total protein. Source of admission was not significantly associated with 90-day post-discharge mortality (HR, 0.831; 95% CI, 0.097–7.096; *p* = 0.865), and its interaction with ln(time) was also not significant (HR, 1.036; 95% CI, 0.573–1.873; *p* = 0.907). Full results of the extended Cox models are presented in Supplementary Tables S2 and S3.

## 4. Discussion

The present study compared nursing-care-dependent older adults admitted to internal medicine departments from home with those admitted from nursing homes. This comparison is clinically important because admission from a nursing home may be considered a marker of poorer prognosis. In health-care systems in which many highly dependent older adults continue to live at home with caregivers, patients admitted from home may not represent a relatively healthy community-dwelling population. Rather, they may have a level of dependency and medical complexity comparable to that of nursing-home residents [1,2,3,4,5,6,7,23,24].

The primary finding was that no statistically significant difference in in-hospital mortality was detected according to source of admission. Similarly, Kaplan–Meier analysis of 90-day post-discharge survival among patients discharged alive did not demonstrate a statistically significant survival difference between the two groups. Notably, despite a higher prevalence of dementia, lower Norton and MNA-mini scores, and more frequent infection-related presentations among patients admitted from nursing homes, these differences were not associated with worse in-hospital or 90-day post-discharge outcomes.

Previous studies have often reported greater clinical vulnerability and poorer outcomes among nursing-home residents than among general community-dwelling older adults, including higher mortality, severe functional limitation, and frequent acute hospital transfer [12,14,15,25,26]. Studies comparing nursing-home residents with community residents found higher mortality and longer hospitalization among nursing-home residents, while population-based studies also showed high rates of acute hospital transfer from nursing homes [14,15,25]. However, some adjusted analyses suggest that the independent association of nursing-home residence with hospital outcomes may be attenuated after accounting for age, diagnosis, comorbidity, and baseline dependency [25]. One possible explanation for the absence of a statistically significant difference in outcomes between nursing-care-dependent older adults admitted from nursing homes and those admitted from home is that nursing-home residents may have access to more structured nursing surveillance and established pathways for hospital referral [15,23,24]. Conversely, less continuous professional monitoring in home-care settings could potentially delay recognition of nonspecific clinical deterioration [23,24]. Overall, findings across studies are inconsistent and may reflect differences in case mix, long-term care structures, and adjustment for baseline frailty.

The present comparison differs from many prior studies because the home group was already nursing-care dependent.

Patients admitted from home were older than those admitted from nursing homes. Older age is associated with reduced physiological reserve and greater vulnerability to hospital-associated complications [10,11]. This age difference may therefore contribute to the overlap in observed outcomes between groups, despite greater dementia, pressure-ulcer risk, nutritional vulnerability, and infection-related presentation among nursing-home patients [10,11,27,28].

The two groups differed in their clinical presentation. Nursing-home patients more often presented with fever, pneumonia, and urinary tract infection, a pattern consistent with the high burden of dementia, immobility, swallowing problems, device use, and infection risk described in long-term-care populations [9,12,14,15,29,30]. Patients admitted from home more often presented with general deterioration and cerebrovascular or other diagnoses. This pattern may reflect delayed recognition of a nonspecific decline in this community, variable availability of professional medical supervision, and the difficulty of identifying acute deterioration in frail patients whose baseline functional status is already poor [23,24].

Comorbidity burden was very high in both groups and did not differ significantly by source of admission. Nearly all participants were in the severe ACCI category [18]. In this highly selected nursing-care-dependent cohort, ACCI may have limited discriminatory spread because severe comorbidity was nearly ubiquitous. This finding is important because it suggests that, in this cohort, the source of admission did not separate two clearly different populations in terms of comorbidity burden. The lack of difference in ACCI may also indicate a ceiling effect: once patients are already nursing-care dependent with multiple chronic illnesses, and the ACCI may have limited ability to distinguish clinically meaningful differences between home-treated and institutionalized patients [18].

The different distribution of selected chronic diseases also supports this interpretation. Diabetes mellitus was more common among patients admitted from home, further supporting the substantial chronic disease burden observed in this group. In contrast, dementia was markedly more common among patients admitted from nursing homes, which is consistent with the established association between cognitive impairment, functional dependence, institutional care, infection risk, and adverse outcomes in older adults [9,12,14,15,26].

Pressure-ulcer-risk and nutritional measures provided information beyond comorbidity. Nursing-home patients had lower Norton scores, indicating greater pressure-ulcer risk. They also had lower MNA-mini scores, indicating greater nutritional vulnerability. The present study did not directly assess whether nutritional monitoring or feeding assistance differed between care settings, so such mechanisms should remain hypotheses rather than data-supported explanations.

Malnutrition, dependency, immobility, and pressure-ulcer risk commonly coexist in hospitalized older adults [19,21,22,31]. In the present cohort, the MNA-mini, but not the numeric MUST score, differed by source of admission. This may reflect differences in the constructs captured by these instruments, but the finding should be interpreted descriptively rather than as evidence that one screening tool is superior [20,21,22].

In this cohort, source of admission was not significantly associated with in-hospital mortality or 90-day post-discharge mortality. In the longer-term all-cause mortality analysis, however, the association between source of admission and mortality varied over time in the extended Cox model. These findings should not be interpreted as evidence of equivalence between the groups. Taken together, these findings suggest that source of admission alone did not clearly distinguish mortality risk in this cohort. Both groups represented highly vulnerable older adults with substantial comorbidity and functional impairment; therefore, mortality risk should be interpreted together with individual clinical characteristics, including function, nutrition, pressure-ulcer risk, cognition, infection status, and laboratory findings [19,20,21,22,23,24,29,30,32]. These findings have several potential clinical implications. Nursing-care-dependent patients admitted from home should not automatically be considered lower risk than nursing-home residents. Infection-related presentations were more frequent among nursing-home patients, whereas patients admitted from home were older and more often presented with general deterioration. The findings highlight the importance of structured community follow-up and coordination among primary care, home-care services, emergency departments, and hospitals for nursing care patients living at home. Differences in community monitoring, referral pathways, or caregiver support are plausible contributors but were not measured and cannot be inferred causally from this retrospective study. Further work should directly evaluate these care-process variables.

This study has several limitations. It was retrospective and conducted at a single center, and the analyses depended on the completeness and accuracy of electronic medical records. A validated frailty instrument was not available, and the Norton scale was used only as a pressure-ulcer-risk measure. Standardized ADL measurements were not consistently available. Some nutritional and laboratory variables were missing, and, consequently, eight patients were excluded from the multivariable all-cause mortality model because of missing covariate data. Analyses restricted to hospital survivors may be subject to survivor-selection bias and should be interpreted separately from analyses beginning at hospital admission. Residual confounding is possible, particularly for acute illness severity, treatment limitations, pre-hospital care intensity, caregiver availability, and other care-process variables not captured in the medical record.

## 5. Conclusions

In conclusion, no statistically significant difference in in-hospital mortality was detected between nursing-care-dependent patients admitted from nursing homes and those admitted from home; this finding does not establish equivalence. Nursing-home patients had a higher prevalence of dementia and greater nutritional, pressure-ulcer-risk, and infection-related vulnerability, whereas patients admitted from home were older. Source of admission alone, therefore, did not clearly distinguish prognosis in this selected cohort. Clinical assessment should remain individualized and include baseline dependency, cognition, nutrition, comorbidity, acute illness severity, and, when available, a validated frailty assessment.

## Figures and Tables

**Figure 1:**
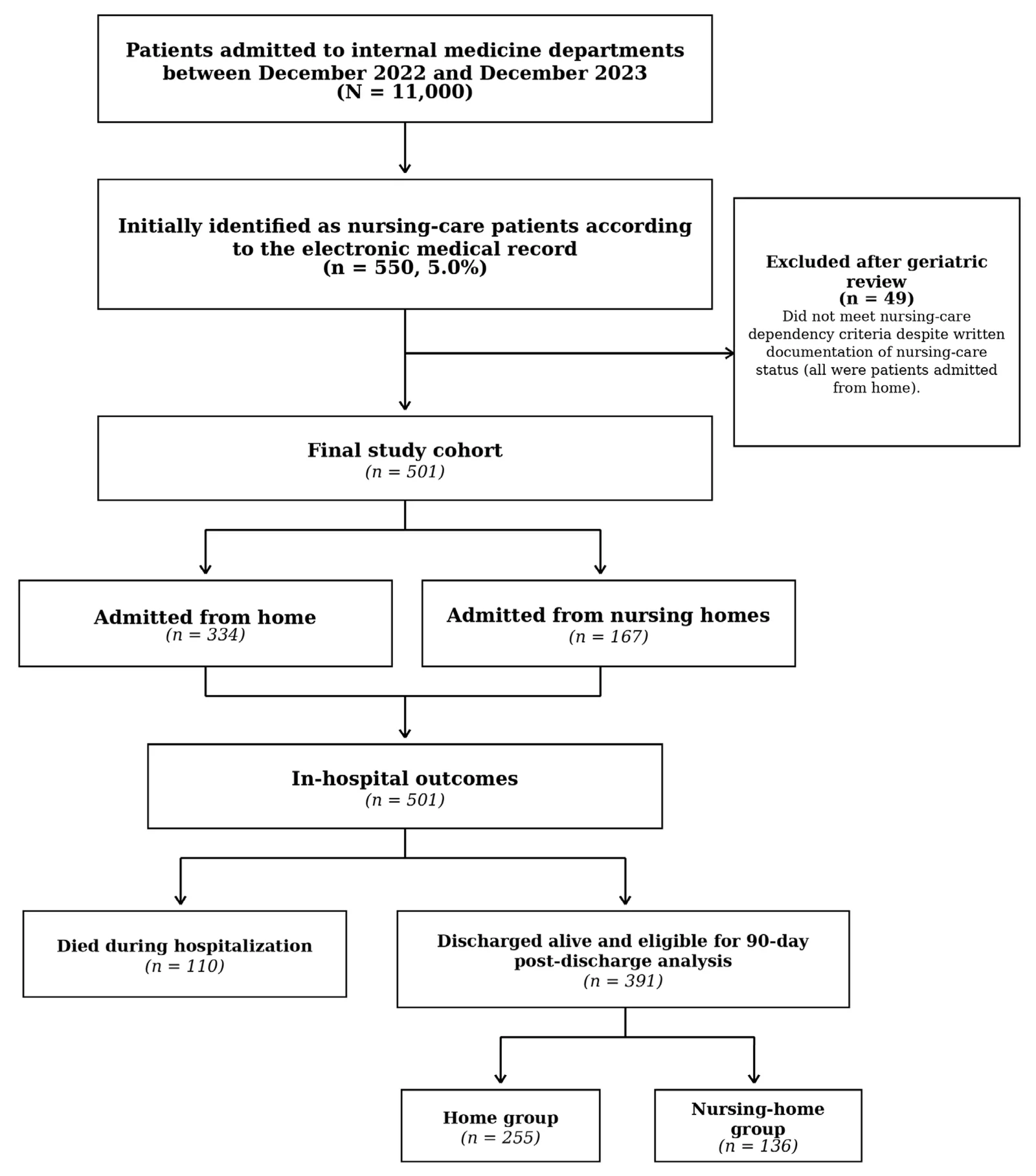
Participant flow diagram showing the numbers of screened, excluded, and included patients and the analytical population for the 90-day post-discharge analysis.

**Figure 2:**
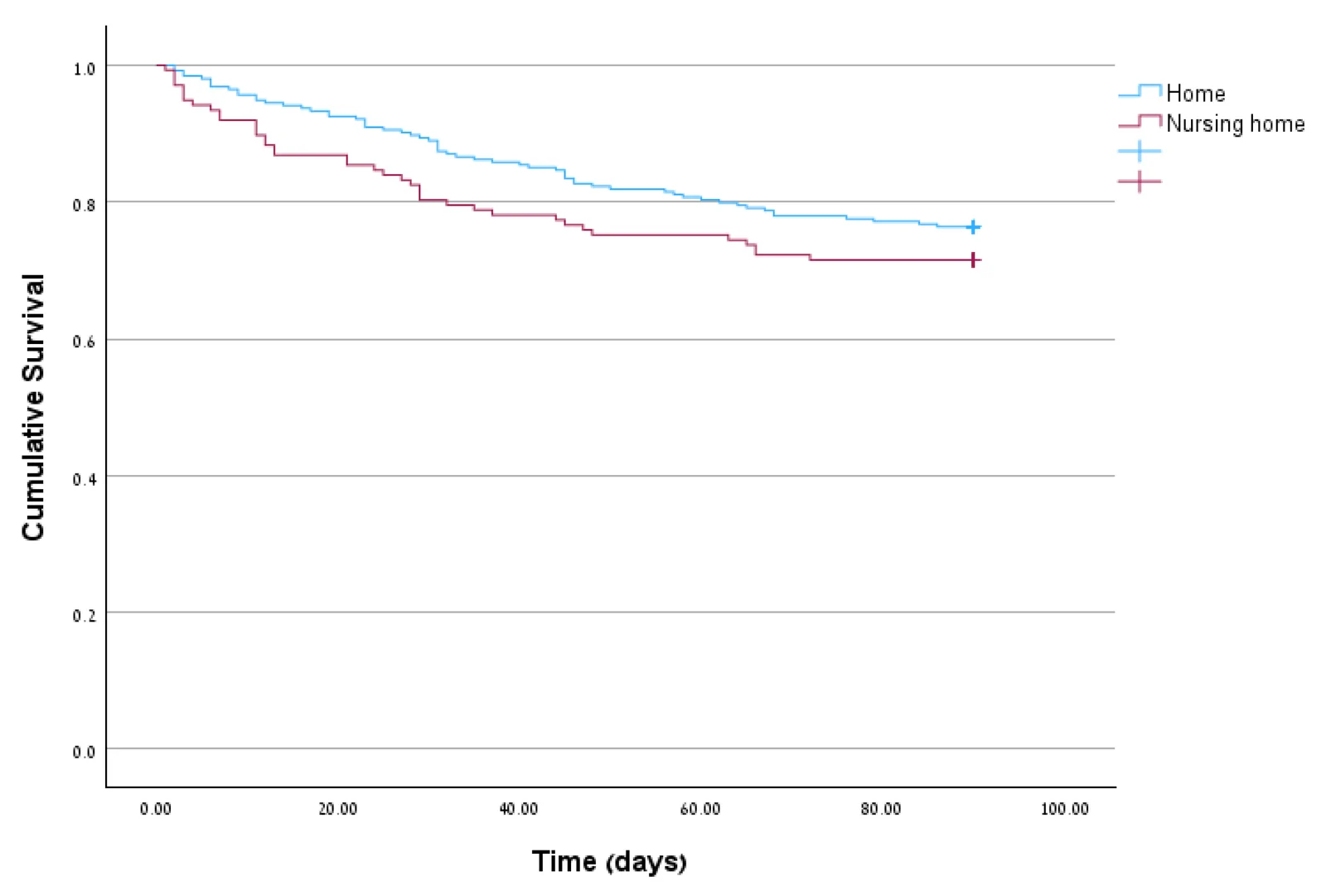
Kaplan–Meier curves for 90-day post-discharge survival by source of admission. Plus signs (+) indicate censored observations.

**Table 1:** Baseline characteristics and admission presentation.

Variable	Total *n* = 501	Home *n* = 334	Nursing Home *n* = 167	*p*-Value
Female sex	316 (63.1%)	220 (65.9%)	96 (57.5%)	0.067
Age, years, median (IQR)	85 (11)	86 (10)	84 (13)	0.036
Main complaint: fever	124 (24.8%)	65 (19.5%)	59 (35.3%)	<0.001
Main complaint: shortness of breath	137 (27.3%)	90 (26.9%)	47 (28.1%)	
Main complaint: confusion	2 (0.4%)	2 (0.6%)	0	
Main complaint: laboratory abnormality	44 (8.8%)	33 (9.9%)	11 (6.6%)	
Main complaint: general deterioration	103 (20.6%)	81 (24.3%)	22 (13.2%)	
Main complaint: other	91 (18.2%)	63 (18.9%)	28 (16.8%)	
Main diagnosis: pneumonia	139 (27.7%)	83 (24.9%)	56 (33.5%)	<0.001
Main diagnosis: UTI	79 (15.8%)	40 (12.0%)	39 (23.4%)	
Main diagnosis: other infection	101 (20.2%)	74 (22.2%)	27 (16.2%)	
Main diagnosis: CVA/TIA	74 (14.8%)	55 (16.5%)	19 (11.4%)	
Main diagnosis: other	108 (21.6%)	82 (24.6%)	26 (15.6%)	
ACCI category: moderate	14 (2.8%)	11 (3.3%)	3 (1.8%)	0.338
ACCI category: severe	487 (97.2%)	323 (96.7%)	164 (98.2%)	
Norton category: moderate	38 (7.6%)	35 (10.5%)	3 (1.8%)	<0.001
Norton category: severe	463 (92.4%)	299 (89.5%)	164 (98.2%)	

Abbreviations: ACCI, Age-adjusted Charlson Comorbidity Index; CVA, cerebrovascular accident; IQR, interquartile range; TIA, transient ischemic attack; UTI, urinary tract infection.

**Table 2:** Comorbidities and risk factors.

Variable	Total *n* = 501	Home *n* = 334	Nursing Home *n* = 167	*p*-Value
Moderate/severe liver disease	13 (2.6%)	8 (2.4%)	5 (3.0%)	0.691
Diabetes mellitus	192 (38.3%)	145 (43.4%)	47 (28.1%)	<0.001
Complicated diabetes	183 (36.5%)	130 (38.9%)	53 (31.7%)	0.115
Hemiplegia	35 (7.0%)	24 (7.2%)	11 (6.6%)	0.804
Moderate/severe CKD	72 (14.4%)	49 (14.7%)	23 (13.8%)	0.787
Solid tumor	84 (16.8%)	61 (18.3%)	23 (13.8%)	0.205
Leukemia	8 (1.6%)	4 (1.2%)	4 (2.4%)	0.313
Metastatic tumor	12 (2.4%)	9 (2.7%)	3 (1.8%)	0.535
Ischemic heart disease	177 (35.3%)	115 (34.4%)	62 (37.1%)	0.552
Congestive heart failure	234 (46.7%)	166 (49.7%)	68 (40.7%)	0.057
Peripheral vascular disease	49 (9.8%)	33 (9.9%)	16 (9.6%)	0.915
CVA/TIA history	201 (40.1%)	132 (39.5%)	69 (41.3%)	0.699
Dementia	336 (67.1%)	194 (58.1%)	142 (85.0%)	<0.001
COPD	88 (17.6%)	57 (17.1%)	31 (18.6%)	0.678
Connective tissue disorder	19 (3.8%)	14 (4.2%)	5 (3.0%)	0.508
Peptic ulcer disease	34 (6.8%)	20 (6.0%)	14 (8.4%)	0.315
Mild liver disease	16 (3.2%)	11 (3.3%)	5 (3.0%)	0.857
Pressure wounds on admission	230 (45.9%)	144 (43.1%)	86 (51.5%)	0.076

Abbreviations: CKD, chronic kidney disease; COPD, chronic obstructive pulmonary disease; CVA, cerebrovascular accident; TIA, transient ischemic attack.

**Table 3:** Pressure ulcer risk, nutritional status, comorbidities, and laboratory measures.

Variable	Total (*n* = 501)	Home (*n* = 334)	Nursing Home (*n* = 167)	*p*-Value
Norton score, median (IQR)	14.0 (4.0)	14.0 (3.0)	11.0 (5.0)	<0.001
MUST score, median (IQR)	0.0 (0.0)	0.0 (0.0)	0.0 (1.0)	0.280
MNA-mini, median (IQR)	6.0 (4.0)	6.0 (3.0)	5.0 (3.0)	0.007
ACCI, median (IQR)	8.0 (3.0)	7.0 (3.0)	8.0 (3.0)	0.886
WBC (K/µL), median (IQR)	10.0 (7.5)	9.5 (6.1)	10.8 (7.7)	0.008
Neutrophil count (K/µL), median (IQR)	7.6 (6.1)	7.4 (5.6)	8.3 (6.9)	0.009
Lymphocyte count (K/µL), median (IQR)	1.2 (1.0)	1.1 (1.0)	1.2 (1.0)	0.123
Hemoglobin (g/dL), median (IQR)	11.2 (3.0)	10.9 (3.0)	11.6 (2.9)	0.053
Hematocrit (%), median (IQR)	33.7 (8.5)	33.5 (8.5)	34.6 (9.2)	0.063
Urea (mg/dL), median (IQR)	63.0 (64.0)	58.1 (65.3)	68.0 (62.0)	0.097
Creatinine (mg/dL), median (IQR)	1.1 (0.9)	1.1 (0.9)	1.1 (1.0)	0.737
Total protein (g/L), median (IQR)	65.0 (9.0)	65.0 (8.0)	66.0 (10.0)	0.165
Albumin (g/L), median (IQR)	32.0 (7.0)	33.0 (7.0)	31.0 (7.0)	0.228
CRP (mg/L), median (IQR)	49.8 (97.0)	45.2 (97.4)	62.6 (101.4)	0.052
Total cholesterol (mg/dL), median (IQR)	136.5 (56.0)	134.0 (58.0)	143.0 (53.0)	0.348
Triglycerides (mg/dL), median (IQR)	102.5 (61.0)	103.0 (60.0)	102.0 (63.0)	0.930

Abbreviations: ACCI, Age-adjusted Charlson Comorbidity Index; CRP, C-reactive protein; MNA-mini, Mini Nutritional Assessment short form; MUST, Malnutrition Universal Screening Tool; WBC, white blood cell count.

**Table 4:** Hospitalization outcomes and mortality.

Outcome	Total	Home	Nursing Home	*p*-Value
In-hospital mortality, *n* (%)	110/501 (22.0%)	79/334 (23.7%)	31/167 (18.6%)	0.194
Length of stay, days, median (IQR)	7.0 (9.0)	7.0 (11.0)	7.0 (9.0)	0.755
Discharge destination among hospital survivors, *n* (%)	*n* = 391	*n* = 255	*n* = 136	0.104
Original location	326 (83.4%)	218 (85.5%)	108 (79.4%)	
Complex nursing care	47 (12.0%)	24 (9.4%)	23 (16.9%)	
Chronic ventilation unit	8 (2.0%)	5 (2.0%)	3 (2.2%)	
Other	10 (2.6%)	8 (3.1%)	2 (1.5%)	
90-day post-discharge mortality among hospital survivors, *n* (%)	101/391 (25.8%)	62/255 (24.3%)	39/136 (28.7%)	0.348
All-cause mortality during follow-up, *n* (%)	406/501 (81.0%)	269/334 (80.5%)	137/167 (82.0%)	0.687

Categorical variables are presented as frequency (percentage). Note: “Other” discharge destination includes rehabilitation and COVID-19 isolation destinations because each category was small. The *p*-value for discharge destination represents the global comparison across categories.

**Table 5:** Kaplan–Meier analysis of 90-day post-discharge survival by source of admission, *n* = 391.

Group	*n*	Events	Censored, *n* (%)	Mean Survival, Days (95% CI)	Median Survival, Days	Log-Rank *p*-Value
Home	255	62	193 (75.7%)	77.2 (74.0–80.4)	Not reached	0.277
Nursing home	136	39	97 (71.3%)	72.7 (67.5–77.9)	Not reached	
Total	391	101	290 (74.2%)	75.6 (72.8–78.4)	Not reached	

Abbreviations: CI = confidence interval.

## Data Availability

The data presented in this study are available from the corresponding author upon reasonable request, subject to institutional and ethical restrictions.

## Supplementary Materials

The following supporting information can be downloaded at: https://www.mdpi.com/article/10.3390/medicina62101880/s1, Figure S1: Kaplan–Meier curve for all-cause survival from hospital admission by source of admission; Table S1: Kaplan–Meier analysis of all-cause survival by source of admission; Table S2: Extended Cox regression analysis for all-cause mortality from hospital admission; Table S3: Extended Cox regression analysis for 90-day post-discharge mortality among hospital survivors.
