# Supplementary material for: Does Source of Admission Matter? Hospital Outcomes of Older Adults Requiring Nursing Care Hospitalized in Internal Medicine Departments
Source: Medicina (Kaunas). 2026 Sep 28;62(10):1880. doi: 10.3390/medicina62101880 (PMC13706921; doi:10.3390/medicina62101880)
Supplement: Supplementary file 1 [file medicina-62-01880-s001.zip › medicina-4549756-supplementary.pdf]

Supplementary Table S1. Kaplan-Meier analysis of all-cause survival from hospital admission by source of admission (N = 501).

Group	N	Events	Censored, n (%)	Mean survival, days (95% CI)	Median survival, days (95% CI)	Log-rank p-value
Home	334	269	65 (19.5%)	414 (367–462)	198 (133–263)	0.183
Nursing home	167	137	30 (18.0%)	350 (289–412)	150 (95–205)	
Total	501	406	95 (19.0%)	396 (357–434)	174 (125–223)	

Abbreviations: *CI = confidence interval.*

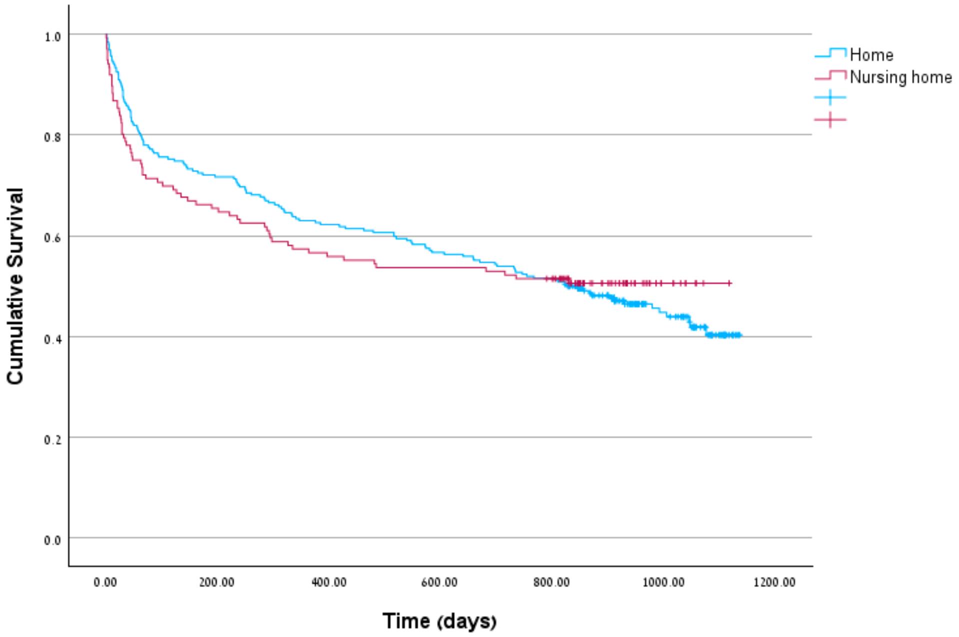

Supplementary Figure S1. Kaplan-Meier curves for all-cause survival from hospital admission by source of admission (N = 501)

Supplementary Table S2. Extended Cox regression model with time-dependent covariates for all-cause mortality from hospital admission.

Model population: N = 493; deaths = 398; censored = 95; 8 patients were excluded because of missing values.

Variable	p-value	HR	95% CI lower	95% CI upper
Age	<0.001	1.441	1.376	1.510
Neutrophil count	<0.001	1.115	1.062	1.171
Urea	0.041	1.006	1.000	1.011
Albumin	<0.001	1.241	1.165	1.322
CRP	0.296	1.003	0.998	1.008
Male sex (vs. female)	0.783	1.104	0.545	2.239
Dementia (yes vs. no)	0.010	0.376	0.179	0.790
Nursing home (vs. home)	<0.001	4.361	2.127	8.940
Age × ln(time)	<0.001	0.926	0.918	0.934
Neutrophil count × ln(time)	<0.001	0.975	0.963	0.986
Urea × ln(time)	0.098	0.999	0.998	1.000
Albumin × ln(time)	<0.001	0.946	0.933	0.959
CRP × ln(time)	0.251	0.999	0.998	1.000
Sex × ln(time)	0.679	0.971	0.844	1.117
Dementia × ln(time)	0.014	1.201	1.037	1.390
Residency × ln(time)	<0.001	0.713	0.616	0.827

Abbreviations: CI, confidence interval; HR, hazard ratio; CRP, C-reactive protein. Home admission was the reference category for source of admission. Time-dependent terms represent covariate × ln(time) interactions.

Supplementary Table S3. Extended Cox regression model with time-dependent covariates for 90-day post-discharge mortality among hospital survivors.

Model population: N = 388; deaths = 100; censored = 288; 3 patients were excluded because of missing values.

Variable	p-value	HR	95% CI lower	95% CI upper
Age	<0.001	1.524	1.365	1.702
Neutrophil count	0.009	1.191	1.044	1.358
Urea	0.455	1.007	0.988	1.027
Lymphocyte count	0.183	0.999	0.998	1.000
Total protein	<0.001	1.207	1.104	1.319
Nursing home (vs. home)	0.865	0.831	0.097	7.096
Age × ln(time)	<0.001	0.891	0.866	0.918
Neutrophil count × ln(time)	0.014	0.956	0.923	0.991
Urea × ln(time)	0.453	0.998	0.992	1.003
Lymphocyte count × ln(time)	0.196	1.000	1.000	1.000
Total protein × ln(time)	<0.001	0.942	0.916	0.969
Residency × ln(time)	0.907	1.036	0.573	1.873

Abbreviations: CI, confidence interval; HR, hazard ratio. Home admission was the reference category for source of admission. Time-dependent terms represent covariate × ln(time) interactions.
